# A novel termini analysis theory using HTS data alone for the identification of *Enterococcus* phage EF4-like genome termini

**DOI:** 10.1186/s12864-015-1612-3

**Published:** 2015-05-28

**Authors:** Xianglilan Zhang, Yahui Wang, Shasha Li, Xiaoping An, Guangqian Pei, Yong Huang, Hang Fan, Zhiqiang Mi, Zhiyi Zhang, Wei Wang, Yubao Chen, Yigang Tong

**Affiliations:** State Key Laboratory of Pathogen and Biosecurity, Beijing Institute of Microbiology and Epidemiology, Beijing, 100071 P.R. China; School of Life Science & Technology, China Pharmaceutical University, 24 Tong Jia Xiang, Nanjing, 210009 P.R. China; Beijing Computing Center, Beijing, 100094 P.R. China

**Keywords:** Termini analysis theory, Antibiotic-resistant *Enterococcus*, Phage, Genome termini

## Abstract

**Background:**

*Enterococcus faecalis* and *Enterococcus faecium* are typical enterococcal bacterial pathogens. Antibiotic resistance means that the identification of novel *E. faecalis* and *E. faecium* phages against antibiotic-resistant Enterococcus have an important impact on public health. In this study, the *E. faecalis* phage IME-EF4, *E. faecium* phage IME-EFm1, and both their hosts were antibiotic resistant. To characterize the genome termini of these two phages, a termini analysis theory was developed to provide a wealth of terminal sequence information directly, using only high-throughput sequencing (HTS) read frequency statistics.

**Results:**

The complete genome sequences of phages IME-EF4 and IME-EFm1 were determined, and our termini analysis theory was used to determine the genome termini of these two phages. Results showed 9 bp 3′ protruding cohesive ends in both IME-EF4 and IME-EFm1 genomes by analyzing frequencies of HTS reads. For the positive strands of their genomes, the 9 nt 3′ protruding cohesive ends are 5′-TCATCACCG-3′ (IME-EF4) and 5′-GGGTCAGCG-3′ (IME-EFm1). Further experiments confirmed these results. These experiments included mega-primer polymerase chain reaction sequencing, terminal run-off sequencing, and adaptor ligation followed by run-off sequencing.

**Conclusion:**

Using this termini analysis theory, the termini of two newly isolated antibiotic-resistant *Enterococcus* phages, IME-EF4 and IME-EFm1, were identified as the byproduct of HTS. Molecular biology experiments confirmed the identification. Because it does not require time-consuming wet lab termini analysis experiments, the termini analysis theory is a fast and easy means of identifying phage DNA genome termini using HTS read frequency statistics alone. It may aid understanding of phage DNA packaging.

## Background

*Enterococcus faecalis* and *Enterococcus faecium* are the most common enterococcal bacteria cultured from humans, making up more than 90 % of clinical isolates [1]. Since the 1990s, *Enterococcus* strains have been found to be opportunistic Gram-positive pathogens, responsible for various diseases [2–8]. Recently, strains of enterococci resistant to vancomycin have emerged, with an increased incidence reported worldwide [9–11]. Vancomycin was one of the last antibiotics to remain reliably effective against enterococci [12]. The continuous overuse and misuse of antibiotics has produced antibiotic-resistant *Enterococcus*. These, most notably vancomycin-resistant *Enterococcus*, have become a threat in nosocomial settings. However, phage therapy has great promise in antibiotic-resistant *Enterococcus* treatment. For this reason, the identification and study of phages for antibiotic-resistant *Enterococcus* may have a significant medical impact in the near future.

The most important information concerning a phage is its genome packaging, which affects its entire life cycle from initiation [13] to viral DNA replication [14], termination, and regulation of transcription [15]. Identifying genome termini identification is crucial to the entire DNA packaging process. High-throughput sequencing (HTS) is an effective means of performing phage genome sequence analysis [16–19], including genome termini analysis. HTS generates a large number of reads. Mining useful sequence information from these large datasets is a key problem in bioinformatics. Conventional methods first use these data to assemble the full sequence of a phage genome and then carry out molecular biology experiments to identify its termini. Unlike these conventional methods, our termini analysis theory can be used to study the phage’s genome termini and genome packaging directly using only the data from the HTS reads. This approach can prove a phage’s termini information without the secondary molecular biological experiments required by conventional methods. It also reduces the time and expense of DNA packaging analysis.

In this study, two newly introduced antibiotic-resistant *Enterococcus* phages, IME-EF4 and IME-EFm1, were analyzed using the current method. By using the termini analysis theory, we easily identified the IME-EF4/IME-EFm1’s termini and hypothesized that the IME-EF4 and IME-EFm1 both have special 9 bp 3′ protruding cohesive ends. Further molecular biological experiments confirmed this hypothesis. This paper is the first to propose the termini analysis theory, which is a means of identifying a phage’s termini without any wet-lab termini conformation experiment.

## Methods

### IME-EF4, IME-EFm1, and their bacterial strains

The lytic *Enterococcus* phages IME-EF4 and IME-EFm1 were isolated from sewage from PLA Hospital 307 (Beijing, China), and the host bacteria for the two phages were isolated from clinical samples in the same hospital. The collaboration between the PLA Hospital 307 and the present laboratory meant that no specific permits were required for these studies. The collected samples were neither privately owned nor protected and did not involve any endangered or protected species.

### IME-EF4/IME-EFm1 isolation

Enrichment cultures were used to isolate IME-EF4 and IME-EFm1 from sewage [20]. Specifically, approximately 2 mL of filtered (Millipore membranes, pore diameter 0.45 μM) sewage was mixed with 2 mL of 3*liquid LB medium and 100 μL *E. faecalis* or *E. faecium* that had been cultured overnight. To amplify the IME-EF4/IME-EFm1, the enrichment culture was incubated at 37 °C for at least 14 h with agitation and then centrifuged (10 min, 10,000 × g, 4 °C). The supernatant was filtered (Millipore membranes, pore diameter 0.45 μM) to remove the residual bacterial cells. Then 100 μL of phage stock solution was mixed with 500 μL EF4/EFm1 cells in the exponential growth phase (OD_600_ = 0.2 to 0.5). The mixture was incubated at 37 °C for 5 min. Then 5 mL top agar (LB with 0.75 % agar) was added at 50 °C, and the mixture was poured into an LB plate that had been pre-warmed to 37 °C (double-layer method). This plate was then incubated overnight at 37 °C to produce phage plaques [21].

### IME-EF4/IME-EFm1 genome DNA extraction

IME-EF4/IME-EFm1 DNA was extracted based on a method described in a previous work [22]. In brief, DNase I and RNase A (Thermo Scientific, America) were added to the phage IME-EF4 stock solution to a final concentration of 1 μg/ml. The mixture was incubated overnight at 37 °C. Then samples were incubated at 80 °C for 15 min to deactivate the DNase I. Lysis buffer (final concentration, 0.5 % sodium dodecyl sulfate, 20 mM EDTA, and 50 μg/ml proteinase K) was added to samples, which were then incubated at 56 °C for 1 h. An equal volume of phenol was added to extract the DNA. After centrifugation at 7000 × g for 5 min, the aqueous layer was removed to a fresh tube containing an equal volume of phenol-chloroform-isoamyl alcohol (25:24:1) and centrifuged at 7000 × g for 5 min. The aqueous layer was collected, mixed with an equal volume of isopropanol, and stored overnight at −20 °C. The mixture was centrifuged at 4 °C for 20 min at 10,000 × g, and the DNA pellet was washed with 75 % ethanol. The DNA was then air dried at room temperature, resuspended in deionized water, and stored at −20 °C.

### IME-EF4/IME-EFm1 high-throughput sequencing

After extraction of the phage IME-EF4 and IME-EFm1 genome DNA, the genomes were using the semiconductor sequencer in the Life Technologies Ion Torrent Personal Genome Machine (PGM) IonTorrent sequencer (IonTorrent). This technology uses emulsion polymerase chain reaction (PCR) and a sequencing-by-synthesis approach [23]. The library preparation, amplification, and sequencing were performed according to the Ion Torrent™ sequencing protocols. The IME-EF4 and IME-EFm1 genome DNA samples were sheared using Ion Shear™ Plus Reagents. These DNA fragments were then ligated to Ion Torrent adapters for subsequent nick repair and purification. Purified DNA fragments of about 300 bp were selected by using E-Gel® SizeSelect™ agarose gel. After amplification and purification of the selected library, emulsion PCR was used to process it. PCR was performed in a water-in-oil microreactor containing a single DNA molecule on a bead [24]. The H^+^ Ion Torrent signal was detected during the sequencing-by-synthesis. In this process, four fluorescently labeled nucleotides were added to the flowcell channels during DNA synthesis. The florescent light signals were detected using a genome analyzer, which was used for base calling [25].

### Termini analysis theory

A phage with a linear double-stranded DNA (dsDNA) has terminal repetitions. These repetitions are used for homologous recombination during the phage’s DNA replication process. The phage’s dsDNA can be circularized through the genome terminal repetitions. This makes the phage’s natural genome termini, which are cleaved by a terminase, difficult to identify. In this study, a termini analysis theory, which can be used to find natural termini using the read frequency, is described.

Suppose that there are *m* identical genomes and the length of each genome is *L*. All the genomes are divided into *N*_*r*_ short sequences. Each short sequence is called a read. The average length of the reads is *L*_*reads*_.

1 $$ \mathbf{Theorem}\ \mathbf{1}\kern0.5em R=\frac{Fre{q}_{ter}}{Fre{q}_{ave}}=2*{L}_{reads} $$

**Fig. 1:**
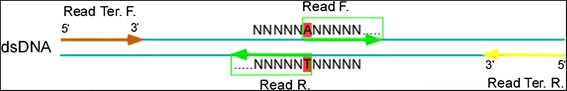
Generation of reads in dsDNA using high-throughput sequencing (HTS). F represents forward sequences and R represents reverse sequences. Ter. refers to termini. The sequence of Read F starts with base A. The sequence of Read R starts with base T. The starting bases are shown with red backgrounds. Reads Ter. F and Ter. R begin with natural termini

*Proof* 1*.* There are *m* identical genomes. As illustrated in Fig. 1, the high-throughput sequencing (HTS) machine reads each read from 5′ to 3′. In this way, each genome with dsDNA has two termini. The frequency of the reads starting with a natural terminus is as follows:

2 $$ Fre{q}_{ter}=m $$

There are a total of *N*_*r*_ reads in the *m* genomes. As shown in Fig. 1, two different reads, F and R, start with base A and base T. In this way, the average frequency of all reads is as follows:

3 $$ Fre{q}_{ave}=\frac{Nr}{2*L} $$

The ratio of *Freq*_*ter*_ to Freq_ave_ is as follows:

4 $$ R=\frac{Fre{q}_{ter}}{Fre{q}_{ave}}=\frac{m}{\frac{Nr}{2*L}}=\frac{2*m*L}{Nr}=2*{L}_{reads} $$

Our termini analysis theory provides a theoretical frequency ratio of terminal reads to general reads. In the experiment, we choose only 300 bp reads rather than the whole amount of reads for analysis. Such a library selection will decrease the practical frequency ratio. Fortunately, we can still practically distinguish the termini from the other reads (see results).

### Mega-primer PCR sequencing

PCR amplification was performed on the IME-EF4 DNA genome to determine more about the IME-EF4 complete genome sequence acquired using the HTS data. The PCR involved mega-primer-guided polymerization through the protruding cohesive end.

### Terminal run-off sequencing

The IME-EF4 complete genome was used as the template for terminal run-off sequencing, the process of which is described in [26]. The 3′ end of the IME-EF4 genome was marked using the primer P1 (5′–CTCTAGTTTGTTGCGTGCGTAAATC–3′). The 5′ end of the IME-EF4 genome was marked using the primer P4 (5′–AGGTACGGACCGCAATGGGTTGGGA–3′). The Sangon Company synthesized the primers (Beijing, China).

### Ligation of the adaptors to the termini

**Fig. 2:**
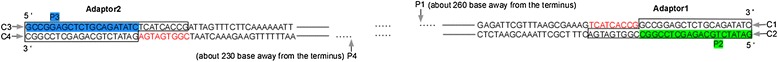
Adaptors and primers used in the experiments. Adaptor 1 includes C1 and C2, and adaptor 2 includes C3 and C4. P2 is highlighted in green, and P3 is highlighted in blue

To prove that IME-EF4 is linear dsDNA ending with a 3′ 9 nt protruding cohesive end, two pairs of adaptors ligated with the ends of IME-EF4 we created. They are shown in Fig. 2. Adaptor 1 was created by combining C1 and C2 and adaptor 2 by combining C3 and C4. To ligate adaptors with IME-EF4 terminal sequences, phosphoric acids were added to C1 and C4. The prepared adaptors are shown in Table 1. At the same time, primers were prepared for use in the next step of PCR amplification, as illustrated in Fig. 2. The primer sequences are shown in Table 2. The Sangon Company prepared the oligonucleotides for these adaptors and primers. The adaptor oligonucleotide mixtures were hybridized by running the ligation program on a PCR machine. To ligate the IME-EF4 genome with the prepared adaptor, phosphoric acid was added for the IME-EF4 genome end repair. Specifically, 800 ng of the IME-EF4 genome was diluted to 16 μL using nuclease-free water, and prepared the end repair mix. Then the phosphate groups were added, also through PCR. The adaptor-ligated DNA was purified as listed in the NEBNext® Fast DNA Library Prep Set for Ion Torrent™ instruction manual (version 3.1). The purified DNA samples were used as templates for PCR amplification and sequencing with the primer in two sets, P1 and P2, and P3 and P4.

**Table 1:** Prepared adaptor list

Adaptor		Sequence (From 5′ to 3′)
Adaptor	C1-P	P-GCCGGAGCTCTGCAGATATC
1	C2	GATATCTGCAGAGCTCCGGC-CGGTGATGA
Adaptor	C3	GCCGGAGCTCTGCAGATATC-TCATCACCG
2	C4-P	P-GATATCTGCAGAGCTCCGGC

**Table 2:** Prepared primer list

Primer	Sequence (From 5′ to 3′)
P1	CTCTAGTTTGTTGCGTGCGTAAATC
P2	GATATCTGCAGAGCTCCGGC
P3	GCCGGAGCTCTGCAGATATC
P4	AGGTACGGACCGCAATGGGTTGGGA

## Results and discussion

### IME-EF4/IME-EFm1 high-throughput sequencing (HTS) analysis

#### IME-EF4/IME-EFm1 high-frequency read statistics

**Fig. 3:**
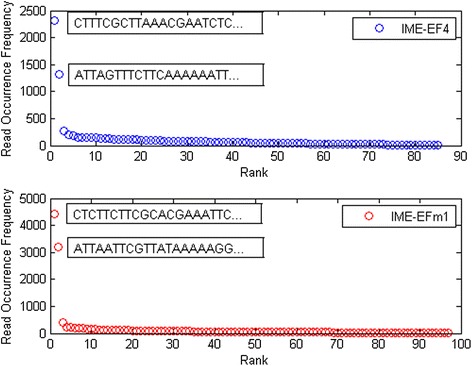
Read occurrence distribution. Blue and red circles indicate the HTS reads of IME-EF4 and IME-EFm1 samples, respectively. The rank in the x-axis refers to the relative frequency of each read

**Fig. 4:**
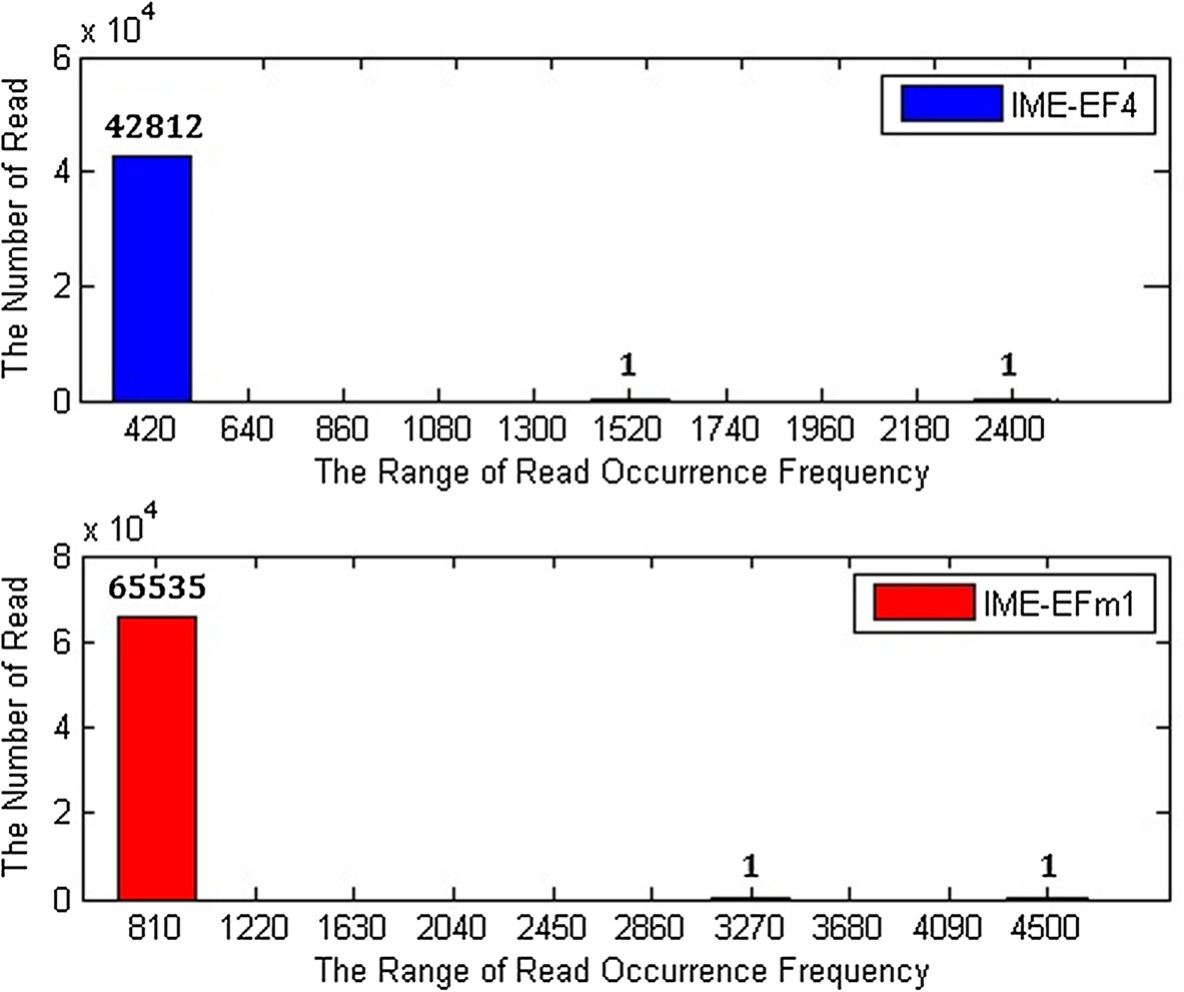
Occurrence rates of numbers of reads. Blue and red bars indicate the HTS reads of IME-EF4 and IME-EFm1 samples, respectively. The number on the x-axis represents the range from the last number to the next number

All HTS reads from the IME-EF4 and IME-EFm1 samples were statistically analyzed and then their frequencies were ranked in descending order. As shown in Fig. 3, IME-EF4 and IME-EFm1 HTS read data have two significant high-frequency reads, beginning with CTTTCGCTTAAACGAATCTC and ATTAGTTTCTTCAAAAAATT, respectively. The HTS data of IME-EF4 and IME-EFm1 share similar sequence occurrence frequency curves. Fig. 4 shows that more than 99 % of reads have frequencies below 420 for IME-EF4 and below 810 for IME-EFm1. Using the termini analysis theory, it was concluded that the two reads with the highest frequencies were the termini of phage IME-EF4 and IME-EFm1. As shown in Table 3, the occurrence rates of terminal reads to general reads were 196 for the positive strand of IME-EF4, 344 for the negative strand of IME-EF4, 246 for the positive strand of IME-EFm1, and 341 for the negative strand of IME-EFm1. Biological experiments were used to verify our conclusion (see behind).

**Table 3:** IME-EF4 and IME-EFm1 terminal sequence frequency statistics

Phage	Strand	Ave. Freq.	Ter. Freq.	$\frac{\mathrm{Ter}.\mathrm{Freq}.}{\mathrm{Ave}.\mathrm{Freq}.}$	Terminal Sequence
IME-EF4	Positive	6.73	1,322	196	ATTAGTTTCTTCAAAAAATT
	Negative	6.73	2,318	344	CTTTCGCTTAAACGAATCTC
IME-EFm1	Positive	12.95	3,194	246	ATTAATTCGTTATAAAAAGG
	Negative	12.95	4,412	341	CTCTTCTTCGCACGAAATTC

#### IME-EF4/IME-EFm1 sequence assembly and identification of termini

The IME-EF4 and IME-EFm1 sequence assembly results were acquired using Newbler (version 2.9, Roche). As shown in Table 4, the genome size of IME-EF4 is 40,713 bp and the read matches percentage was more than 97 %, while the genome size of IME-Efm1 is 42,599 bp and the read matches percentage was more than 95 %.

**Table 4:** IME-EF4 and IME-EFm1 sample sequence assembly results

Phage	Length	# Matched Reads	# Total Reads	Relative Matches	Min Coverage	Max Coverage
IME-EF4	40,713	548,309	563,429	97.32%	11	7,215
IME-EFm1	42,599	1,103,439	1,154,818	95.55%	51	9,657

**Fig. 5:**
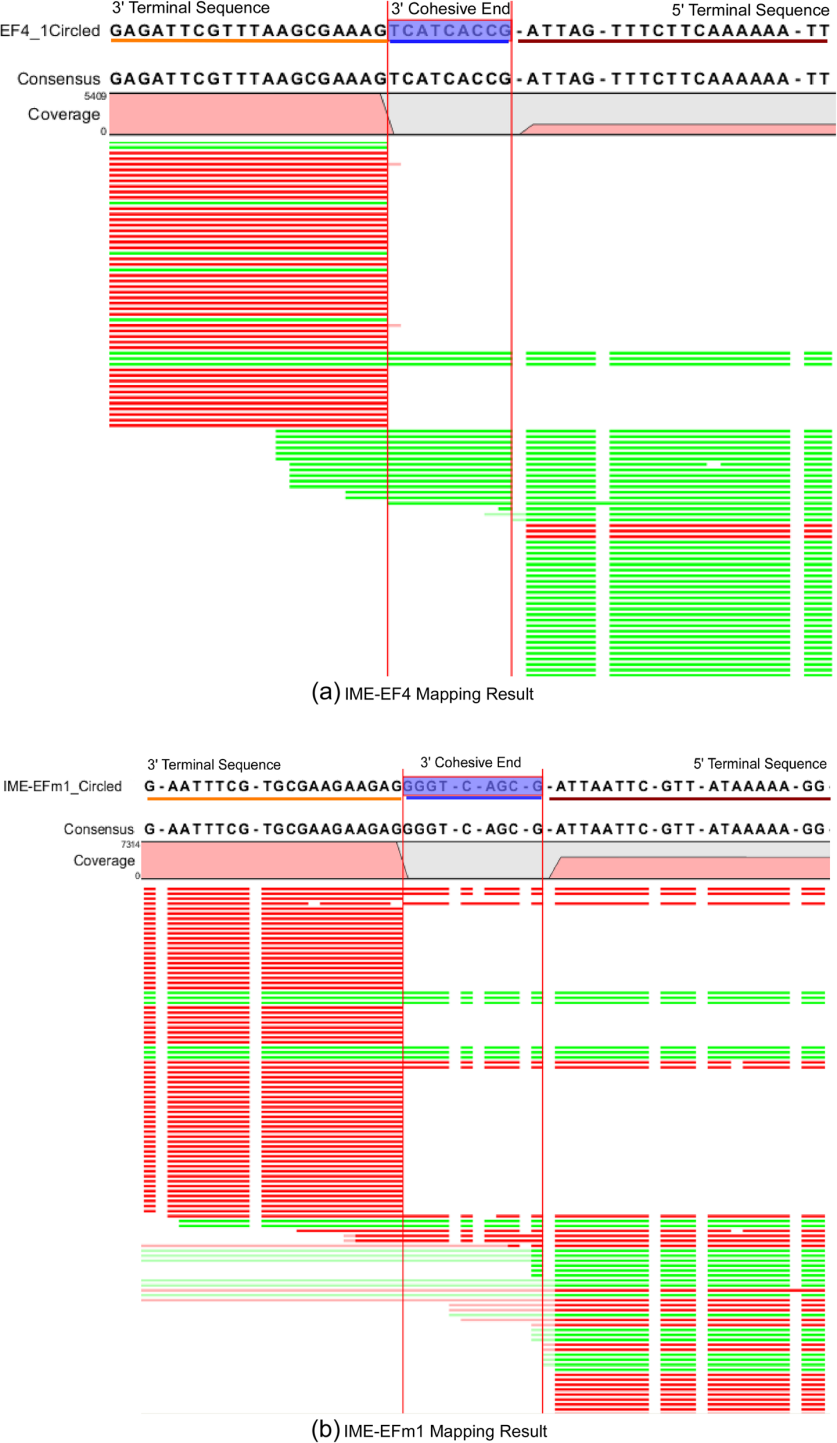
**(a)** IME-EF4 and **(b)** IME-EFm1 mapping results. The mapped reads were acquired from the original HTS data. The 3′ terminal sequences are underlined in orange, the 5’ terminal sequences are underlined in dark red, and the 3′ protruding cohesive ends are underlined in blue

**Fig. 6:**
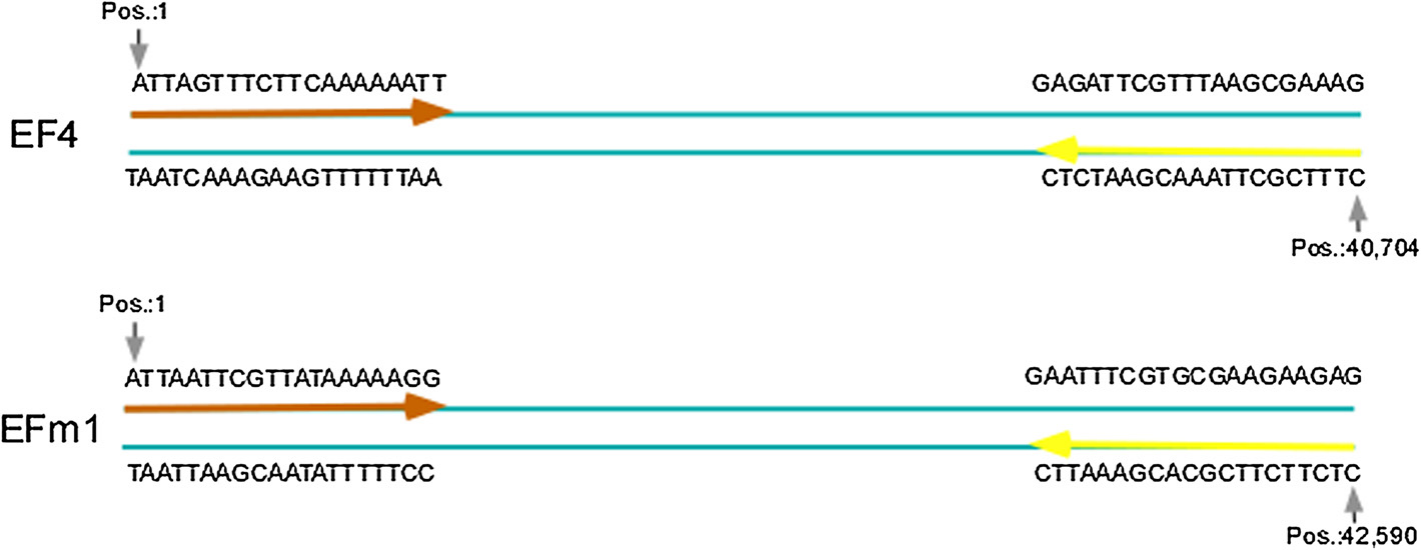
Terminal sequences and their positions. Pos. refers to base positions. The dark orange arrows indicate the 5’ terminal sequences in HTS read data, and the light yellow arrows indicate the 3’ terminal sequences in HTS read data

The reads were then aligned to the reference assembly using a CLC Genomics Workbench (version 3.6.1). The CLC module was run with default parameters (mismatch cost 2, insertion cost 3, deletion cost 3, length fraction 0.5, and similarity 0.8) using FASTQ files as read input and using FNA files as reference input. The results are shown in Fig. 5. The reference genomes are indexed as IME-EF4_Circled and IME-EFm1_Circled, respectively. For IME-EF4, the sequence TCATCACCG is the 9 nt tail that connects the 5′ and 3′ ends. For IME-EFm1, the sequence GGGTCAGCG is the 9 nt tail that connects 5′ and 3′ ends. Two 5′ ends start with ATTAGTTTCTTCAAAAAATT (IME-EF4) and ATTAATTCGTTATAAAAAGG (IME-EFm1). Two 3′ ends start with GAGATTCGTTTAAGCGAAAG (IME-EF4) and CTCTTCTTCGCACGAAATTC (IME-EFm1). Fig. 6 shows the positions of terminal sequences in IME-EF4 and IME-EFm1, each with a 9 nt protruding cohesive end. The last bases of the 3′ termini were located at position 40,704 (IME-EF4, position = genome size (40,713) − protruding cohesive end size (9)) and 42,590 (IME-EFm1, position = genome size (42,590) − protruding cohesive end size (9)).

**Fig. 7:**
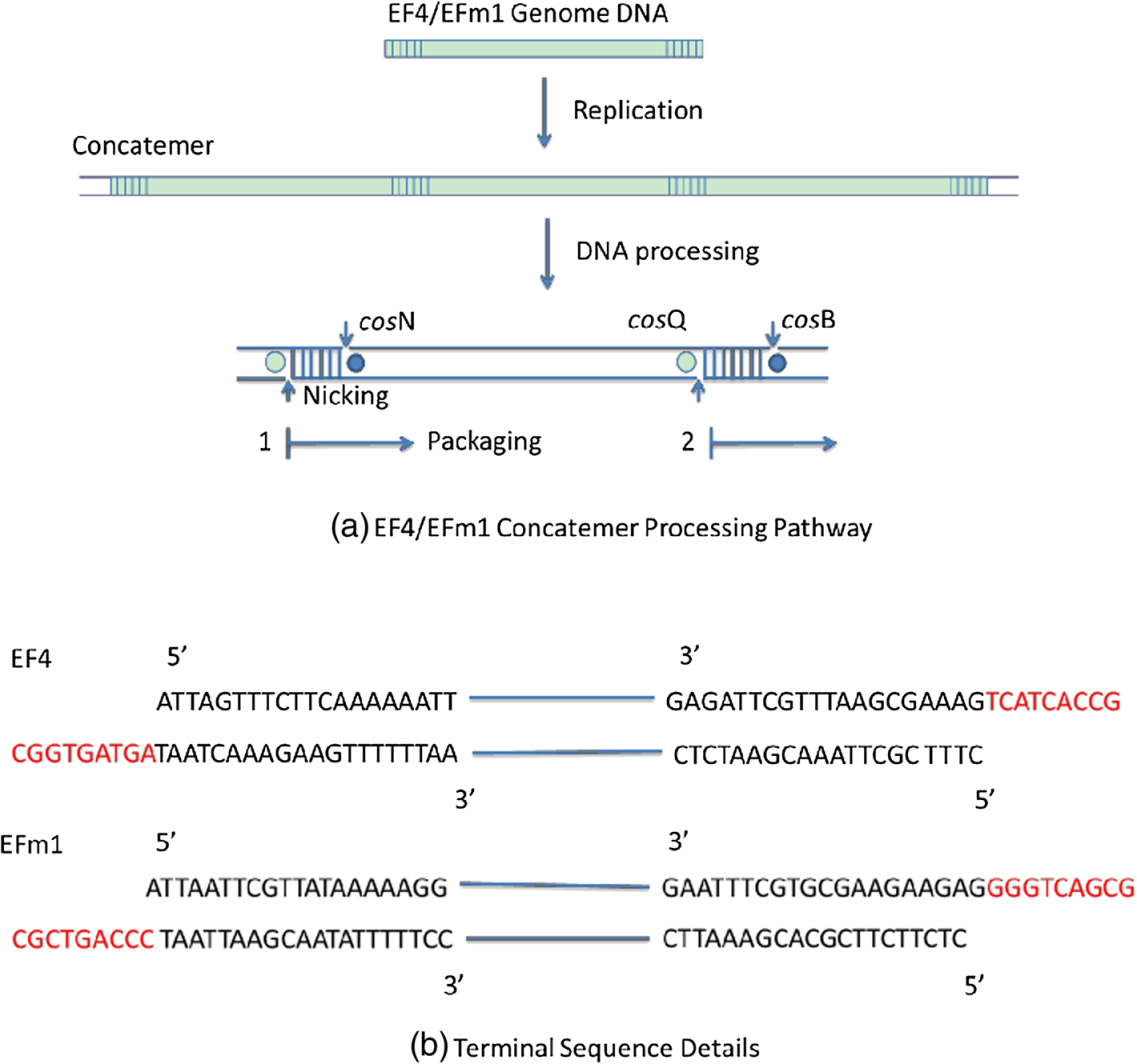
**(a)** IME-EF4 and IME-EFm1 concatemer processing pathways and **(b)** terminal sequence details. The packaging of concatemeric dsDNA is initiated from the end generated by the initiation cleavage, which then proceeds unidirectionally before being terminated by the termination cleavage. IME-EF4/IME-EFm1 terminases introduce nicks staggered by 9 bp concatemer (highlighted in red) at *cos*N and melts the strands by its helicase activity after recognition of *cos*B (●)and *cos*Q (O) for initiation and termination cleavage, respectively

As shown in Fig. 5, there were fewer than 20 cases of short reads with the 9 nt protruding cohesive end. As shown in Table 3 and Fig. 3, the 9 nt protruding cohesive end was not detected in the most frequently occurring reads. Considering that HTS generates the dsDNA reads, it is possible that mature phage IME-EF4 and IME-EFm1 DNA molecules are the ones that have the 9 nt single-stranded 3′ ends, which are called protruding cohesive ends. The reason is explained as follows. If a terminase introduces a double-stranded break into the cos site of the IME-EF4/IME-EFm1, the 5′ and 3′ terminal sequences should ligate to each other without any internal sequence between them. However, this does not match the results of the present assembly experiments, as shown in Fig. 5. In another assumption, the IME-EF4/IME-EFm1 had a 5′ protruding cohesive end. Because the HTS experiment uses the T4 DNA polymerase and because T4 DNA polymerase catalyzes the synthesis of DNA in the 5′- > 3′ direction with 3′- > 5′ exonuclease activity rather than with 5′- > 3′ exonuclease function, the 5′ protruding cohesive end must be repaired. This means that a repeated sequence between the 5′ and 3′ terminal sequences should be detectable. However, there were few short reads including both 5′ and 3′ terminal sequences in the HTS data. Since the linear phage genome was circularized through the genome terminal repetitions during the replication of phage genome, the short reads including 5’ and 3’terminal sequences found in the HTS data might be the intermediate form of the circularized linear phage genome. Therefore, IME-EF4 and IME-EFm1 may both have 9 bp 3′ protruding cohesive ends. As shown in Fig. 7, the terminase cleaves the IME-EF4/IME-EFm1 dsDNA from cosB and cosQ, which has a 9 nt protruding cohesive end between them.

### Experimental verification

To confirm the conclusions drawn from the HTS data analysis, three molecular biology experiments were performed on IME-EF4. These experiments showed that the high-frequency reads were indeed the termini, which means that this termini analysis theory can be used to locate the phage’s termini and their positions using only HTS data even if the procedure is performed quickly.

#### Mega-primer PCR sequencing

**Fig. 8:**
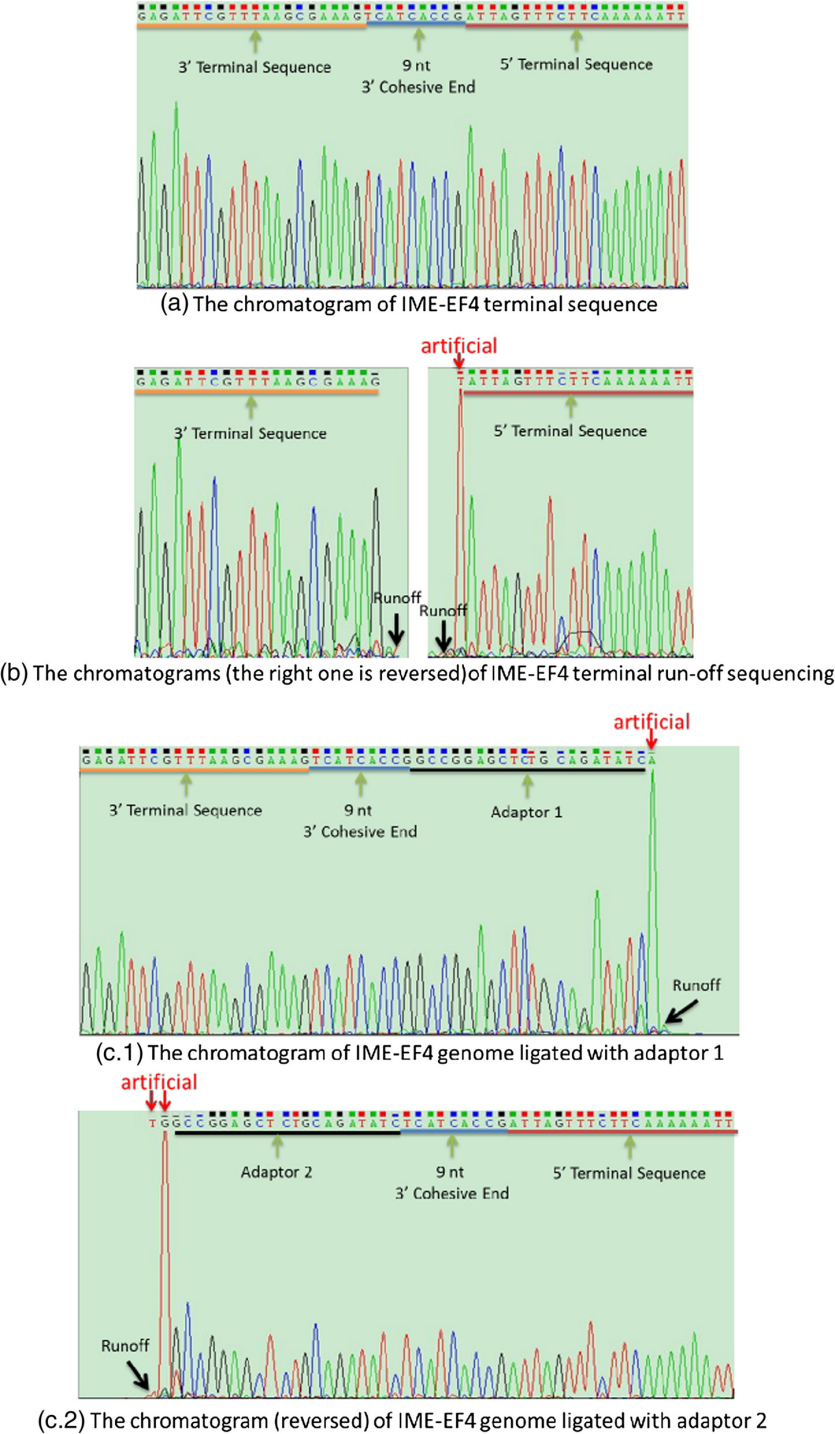
Chromatograms of the three molecular biology experiments, including **(a)** IME-EF4 complete genome sequencing, **(b)** IME-EF4 terminal run-off sequencing, and **(c)** IME-EF4 adaptor ligation to the terminal sequences

To identify the IME-EF4 complete genome sequence acquired by the HTS data, PCR amplification was performed on the IME-EF4 DNA genome. The PCR involved mega-primer-guided polymerization through the protruding cohesive end. The genome sequence snapshot including the 9base is shown in Fig. 8(a), where the upstream sequence was …GAGATTCGTTTAAGCGAAAG and the downstream sequence was ATTAGTTTCTTCAAAAAATT… The results of PCR were consistent with those of HTS data statistical analysis (Fig. 5(a)). It proves that both the IME-EF4 complete genome and the protruding cohesive ends acquired from the HTS data statistics are correct.

#### Terminal Run-off sequencing

**Fig. 9:**
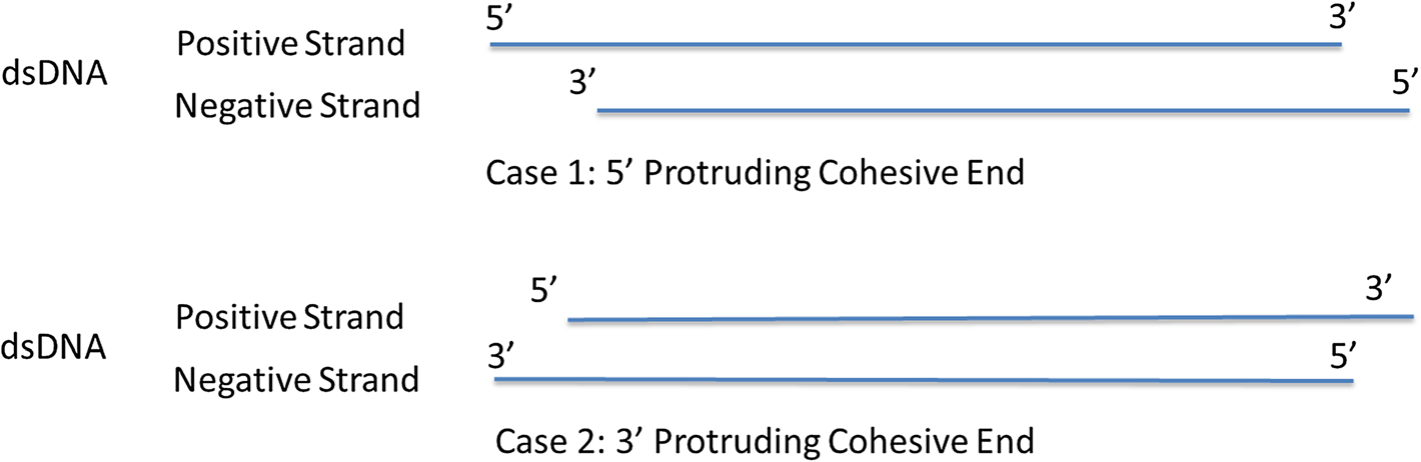
Hypothetical genome dsDNA protruding cohesive end situations

As shown in Fig. 9, two different cases of dsDNA protruding cohesive ends hypothetically exist. Case 1 shows the 5′ protruding cohesive end situation, and case 2 describes the putative IME-EF4 situation of the 3′ protruding cohesive end. If the IME-EF4 has the protruding cohesive end situation according to cases 1, then there would be signals after either the terminal sequence GAGATTCGTTTAAGCGAAAG, ATTTTTTGAAGAAACTAATA, or both of them in the terminal run-off sequencing result. However, as shown in Fig. 8(b), no signal was detected after the termini GAGATTCGTTTAAGCGAAAG in the positive strand or after the termini ATTTTTTGAAGAAACTAATA in the negative strand. These results confirmed the conclusion that IME-EF4 has a linear, double-stranded DNA genome with a 9 nt 3′ protruding cohesive end, represented by case 2–the 3′ protruding cohesive end situation.

#### Adaptor ligation to the termini

**Fig. 10:**
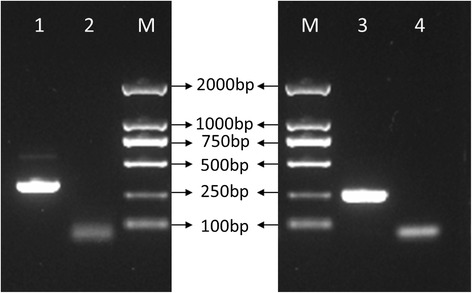
Agarose gel electrophoresis to confirm adaptor ligation. “M” represents marker, “1” is the PCR result of the 3′ IME-EF4 genome sequence ligated with adaptor 1 (primer P1 and P2), “3” is the PCR result of the 5′ IME-EF4 genome sequence ligated with adaptor 2 (primer P3 and P4), and “2” and “4” are the negative control PCR results of the 3′ and 5′ IME-EF4 genome sequences without any adaptor

As shown in Fig. 10, the 3′ IME-EF4 genome sequence ligated to adaptor 1 was about 280 bp in size, and 5′ IME-EF4 genome sequence ligated to adaptor 2 was about 250 bp in size. These findings were consistent with experimental design (P1 and P2 illustrations in Fig. 2). The sequencing results are shown in Fig. 8(c). This further proves that the two adaptors (adaptor 1 and adaptor 2) had successfully ligated to the IME-EF4 genome, which means the IME-EF4 genome has a 9 bp 3′ protruding cohesive end.

## Conclusions

In this study, using a novel termini analysis theory, the complete genome sequences and termini of newly isolated antibiotic-resistant *E. faecalis* phage IME-EF4 and antibiotic-resistant *E. faecium* phage IME-EFm1 were extracted and identified. Analysis of the IME-EF4/IME-EFm1’s HTS data indicated that both IME-EF4 and IME-EFm1 have 9 bp 3′ protruding cohesive ends (TCATCACCG in IME-EF4 positive strand, and GGGTCAGCG in IME-EFm1 positive strand). Further molecular biological experiments, including mega-primer PCR sequencing, terminal run-off sequencing, and adaptor ligation to the termini, fully supported these conclusions. Results indicated that this termini analysis theory facilitated acquisition of important phage genome termini information through analysis of HTS data alone, with no further molecular biology experiments required. In addition, the current analysis of the newly isolated antibiotic-resistant *E. faecalis* and antibiotic-resistant *E. faecium* phages, IME-EF4 and IME-EFm1, have enriched the knowledge of antibiotic-resistant *Enterococcus* phages, which is essential to future antibiotic-resistant *Enterococcus* phage therapy.

### Availability of supporting data

The data sets supporting the results of this article are available in the NCBI GenBank repository, [IME-EF4 complete genome http://www.ncbi.nlm.nih.gov/nuccore/589892984?report=genbankandhttp://www.ncbi.nlm.nih.gov/nuccore/641468964].
